# Asymmetric Bimodal Exponential Power Distribution on the Real Line

**DOI:** 10.3390/e20010023

**Published:** 2018-01-03

**Authors:** Mehmet Niyazi Çankaya

**Affiliations:** 1 Department of International Trading, School of Applied Sciences, Uşak University, Uşak 64200, Turkey; mehmet.cankaya@usak.edu.tr; Tel.: +90-276-221-21-21 (ext. 7051); 2 Department of Statistics, Faculty of Arts and Sciences, Uşak University, Uşak 64200, Turkey

**Keywords:** asymmetric bimodality, bimodal exponential power distribution, modelling, generalized Gaussian distribution

## Abstract

The asymmetric bimodal exponential power (ABEP) distribution is an extension of the generalized gamma distribution to the real line via adding two parameters that fit the shape of peakedness in bimodality on the real line. The special values of peakedness parameters of the distribution are a combination of half Laplace and half normal distributions on the real line. The distribution has two parameters fitting the height of bimodality, so capacity of bimodality is enhanced by using these parameters. Adding a skewness parameter is considered to model asymmetry in data. The location-scale form of this distribution is proposed. The Fisher information matrix of these parameters in ABEP is obtained explicitly. Properties of ABEP are examined. Real data examples are given to illustrate the modelling capacity of ABEP. The replicated artificial data from maximum likelihood estimates of parameters of ABEP and other distributions having an algorithm for artificial data generation procedure are provided to test the similarity with real data. A brief simulation study is presented.

## 1. Introduction

The different bimodal and skew distributions have been proposed over the last decade to construct flexible distributions. The proposed distributions are in [1,2,3,4,5,6,7,8,9,10,11,12,13,14,15,16,17,18,19,20,21,22,23,24,25,26] and references therein via using different generating techniques [27] to get a probability density function (PDF). In these distributions, ε-skew form of gamma distribution on the real line was proposed by [5,6]. The deficiency of these functions is that different height and shape of peakedness around location on the real line cannot be modelled separately. The model proposed by [6] has a bimodality with the same height, which is not flexible enough to model bimodal data with different height and shape of peakedness. The bimodal and alpha-skew Laplace distribution that does not model shape peakedness around location on the real line was proposed by [24]. However, the best way is to find a function that can fit data around location separately. In other words, the left and right sides of location will be modelled with different parameters to have an efficient fitting for both sides of the location. A bimodal exponential power (BEP) distribution is proposed by [28]. The properties of BEP distribution are few when BEP is compared with distribution proposed by [29] because BEP has the same level of peaks around location on the real line, and it is also symmetric on both sides of the location. The shape of peakedness around location on the real line is modelled by only one parameter; however, two parameters are added in order to model different modes from distribution on the real line [29]. Two parameters controlling fitting the shape of peakedness and two parameters controlling fitting the height of bimodality will be used together. Skewness parameter is also added to model asymmetry in data. Thus, modelling capacity of asymmetric bimodal exponential power (ABEP) distribution is better than current candidates proposed by [5,6,28,29] because ABEP distribution has parameters that control the fitting both sides of location separately.

The second aim is that we do not only propose ABEP distribution but also derive this distribution via constructing a normalizing constant (NC), which leads to producing a PDF. While deriving a PDF, producing NC can be a preferable approach. This approach can be taken care for deriving a PDF when one wants to add a new parameter to increase the modelling capacity of function if it is tractable to get NC from a function. The NC approach was examined by [30] to construct asymmetric distributions from symmetric distributions. Some techniques used to derive a PDF are reviewed by [27]. There are other techniques to produce PDFs derived from entropy functions via the method of Lagrange multipliers as well [31,32] and references therein. The different goodness of fit tests (GOFTs) are applied to the ABEP. Thus, importance and advantage of GOFTs, such as Kolmogorov–Smirnov (KS), Cramér von Mises (CVM), Anderson–Darling (AD) via a cumulative distribution function (CDF) of a PDF will be expressed for ABEP distribution when the optimization problem of ABEP can arise.

In particular, the estimation of location parameter is important—for example, the proteins in cancer cells need to be determined, and the image processing demands for obtaining the quantitative value of colors at a prescribed range. A radar data, speech processing, etc. in many phenomena can be modelled via ABEP. The parametric models that can accommodate the shape of peakedness, bimodality and skewness are mostly preferred to be able to model the data set efficiently. In other words, the frequented data can be represented by the parameters that control fitting the shape of peakedness, the parameters that control fitting the bimodality and the skewness that controls fitting the asymmetry in the data set. Due to this reason, ABEP distribution having these parameters is proposed. In addition, since the generalized gamma distribution is a class for many distributions, it is chosen in order to reflect to the negative side of the real line.

The paper is organized as follows. In Section 2, ABEP distribution is defined and mode, distributional properties, related distributions and tail behaviour of ABEP distribution are given. Maximum likelihood (ML) estimations of parameters are provided in Section 3. A brief simulation study is given in Section 4. In Section 5, the real data examples are provided to make a comparison among candidate densities. The results are commented. Finally, in the last section, conclusions are given and remarks are considered.

## 2. Gamma Distribution: Reparametrization and ABEP Distribution on Real Line

The random variable *Y* will have a gamma distribution with PDF having parameters $\frac{\delta +1}{\alpha }$:

(1) $$g\left(y\right)=\frac{1}{\Gamma (\frac{\delta +1}{\alpha })}y^{\frac{\delta +1}{\alpha }-1}\exp\left\{-y\right\},\;\;\;\;y>0,\;\delta >0,\;\alpha >0.$$

**Theorem** **1.** *Let Y be a continuous random variable defined on [0,∞), distributed as $G(\frac{\delta +1}{\alpha })$. Consider a discrete random variable T, which generates a function on the real line. Then, unequal probabilities at negative and positive sides of the real line will be constructed. T is 1+ε with the probability $\frac{1+\varepsilon }{2}$ on the positive side and T is -(1-ε) with the probability $\frac{1-\varepsilon }{2}$ on the negative side. A variable transformation $Z=Y^{1/\alpha }T$ is applied to get the α power of Gamma distribution. Here, the random variables Y and T are independent* [5,29]*. After applying this transformation on gamma distribution in Equation (1), we will get the following PDF:*

(2) $$f\left(z\right)=\left\{\begin{array}{cc} f_{1}\left(z\right)=\frac{\alpha }{2{\left(1-\varepsilon \right)}^{\delta }\Gamma \left(\frac{\delta +1}{\alpha }\right)}{\left(-z\right)}^{\delta }\exp\left\{-{\left(\frac{-z}{1-\varepsilon }\right)}^{\alpha }\right\}, & z<0,\\ f_{0}\left(z\right)=\frac{\alpha }{2{\left(1+\varepsilon \right)}^{\delta }\Gamma \left(\frac{\delta +1}{\alpha }\right)}z^{\delta }\exp\left\{-{\left(\frac{z}{1+\varepsilon }\right)}^{\alpha }\right\}, & z\geq 0, \end{array}\right.$$

*where the parameters α>0,δ>0 and ε∈(-1,1)* [29]. *The random variable T keeps being PDF, which will be generated because the gamma distribution has a PDF defined at the interval [0,∞). The probabilities of (1+ε) and -(1-ε) values of random variable T are $\frac{1+\varepsilon }{2}$ and $\frac{1-\varepsilon }{2}$, respectively* [30,33]. *Thus, a function in Equation (2) has the unequal probabilities on positive and negative sides of the real line. The following PDF from a function in Equation (2) will be proposed:*

(3) $$f\left(z\right)=\left\{\begin{array}{cc} f_{1}\left(z\right)=\frac{\alpha_{1}}{2{\left[k_{1}\left(1-\varepsilon \right)\right]}^{\delta_{1}+1}\Gamma \left(\frac{\delta_{1}+1}{\alpha_{1}}\right)}{\left(-z\right)}^{\delta_{1}}\exp\left\{-{\left(\frac{-z}{k_{1}\left(1-\varepsilon \right)}\right)}^{\alpha_{1}}\right\}, & z<0,\\ f_{0}\left(z\right)=\frac{\alpha_{0}}{2{\left[k_{0}\left(1+\varepsilon \right)\right]}^{\delta_{0}+1}\Gamma \left(\frac{\delta_{0}+1}{\alpha_{0}}\right)}z^{\delta_{0}}\exp\left\{-{\left(\frac{z}{k_{0}\left(1+\varepsilon \right)}\right)}^{\alpha_{0}}\right\}, & z\geq 0, \end{array}\right.$$

*where the parameters $\alpha_{1}>0,\alpha_{0}>0,\delta_{1}>0,\delta_{0}>0,k_{1}>0,k_{0}>0$ and ε∈(-1,1). Without consulting the variable transformation technique, PDF can be obtained. This PDF is called an asymmetric bimodal exponential power distribution (ABEP). $\alpha_{1}$ and $\alpha_{0}$ are for the shape of peakedness, $\delta_{1}$ and $\delta_{0}$ are for height of bimodality on negative and positive sides of the real line. $k_{1}$ and $k_{0}$ are nuisance parameters to have the same form of normal or Laplace distributions. ε is a skewness parameter that is responsible for having unequal probabilities at negative and positive sides of the real line. Thus, a skewness on a function can be constructed. The details for functions in Equation (3) are given by the following proof.*

**Proof.**  The preliminary tools for the calculation of integrals are required. The gamma function and the incomplete gamma functions are used to have integral kernels, which are appropriate for calculating the integrals. Thus, we can derive a PDF:

(4) Γ(s)=γ(s,α)+Γ(s,α),

where $\Gamma \left(s\right)=\int_{0}^{\infty }x^{s-1}\exp\left\{-x\right\}dx$, $\gamma \left(s,\alpha \right)=\int_{0}^{\alpha }x^{s-1}\exp\left\{-x\right\}dx$, and $\Gamma \left(s,\alpha \right)=\int_{\alpha }^{\infty }x^{s-1}\exp\left\{-x\right\}dx$. These are the gamma, the lower and upper incomplete gamma functions, respectively [34].

The reparametrization of gamma function is considered as:

(5) $$\Gamma \left(s+1/\alpha \right)=\int_{0}^{\infty }x^{s+1/\alpha -1}\exp\left\{-x\right\}dx.$$

A variable transformation $x={\left(yp\right)}^{\alpha }$ is applied to get the power version of gamma function:

(6) $$\Gamma \left(s+1/\alpha \right)=\alpha p^{\alpha s+1}\int_{0}^{\infty }y^{\alpha s}\exp\left\{-{\left(yp\right)}^{\alpha }\right\}dy.$$

From Equation (4), $\gamma \left(s^{*},\alpha^{*}\right)=\Gamma \left(s^{*}\right)-\Gamma \left(s^{*},\alpha^{*}\right)$. Now, let $s^{*}$ be s+1/α and $\alpha^{*}={\left(pk\right)}^{\alpha }$. Then, $\gamma \left(s+1/\alpha ,{\left(pk\right)}^{\alpha }\right)=\int_{0}^{{\left(pk\right)}^{\alpha }}x^{s+1/\alpha -1}\exp\left\{-x\right\}dx$. Now, the variable transformation $x={\left(yp\right)}^{\alpha }$ is applied to the power version of the lower incomplete gamma function:

(7) $$\gamma \left(s+1/\alpha ,{\left(pk\right)}^{\alpha }\right)=\alpha p^{\alpha s+1}\int_{0}^{k}y^{\alpha s}\exp\left\{-{\left(yp\right)}^{\alpha }\right\}dy.$$

From Equation (4), $\Gamma \left(s^{*},\alpha^{*}\right)=\Gamma \left(s^{*}\right)-\gamma \left(s^{*},\alpha^{*}\right)$. Now, let $s^{*}$ be s+1/α and $\alpha^{*}={\left(pk\right)}^{\alpha }$. Then, $\Gamma \left(s+1/\alpha ,{\left(pk\right)}^{\alpha }\right)=\int_{{\left(pk\right)}^{\alpha }}^{\infty }x^{s+1/\alpha -1}\exp\left\{-x\right\}dx$. Now, the variable transformation $x={\left(yp\right)}^{\alpha }$ is applied to the power version of the upper incomplete gamma function:

(8) $$\Gamma \left(s+1/\alpha ,{\left(pk\right)}^{\alpha }\right)=\alpha p^{\alpha s+1}\int_{k}^{\infty }y^{\alpha s}\exp\left\{-{\left(yp\right)}^{\alpha }\right\}dy.$$

Equations (6)–(8) are power versions of gamma functions defined on the positive axis. These three functions can be transferred to the negative axis via the variable transformation y=-u. For Equation (6),

(9) $$\Gamma \left(s+1/\alpha \right)=\alpha p^{\alpha s+1}\int_{-\infty }^{0}{\left(-u\right)}^{\alpha s}\exp\left\{-{\left(-up\right)}^{\alpha }\right\}du.$$

For Equation (7),

(10) $$\gamma \left(s+1/\alpha ,{\left(pk\right)}^{\alpha }\right)=\alpha p^{\alpha s+1}\int_{-k}^{0}{\left(-u\right)}^{\alpha s}\exp\left\{-{\left(-up\right)}^{\alpha }\right\}du.$$

For Equation (8),

(11) $$\Gamma \left(s+1/\alpha ,{\left(pk\right)}^{\alpha }\right)=\alpha p^{\alpha s+1}\int_{-\infty }^{-k}{\left(-u\right)}^{\alpha s}\exp\left\{-{\left(-up\right)}^{\alpha }\right\}du.$$

For two cases of x<0 and x≥0, we have the integrals of Equation (3). Hence, Equations (6) and (9) can be used to calculate these integrals. One can easily show that the integrated values of negative and positive sides of Equation (3) are 1/2, respectively. Due to the fact that we must have a PDF defined on the real line, the summation of these two results is 1. Here, the variable transformation technique is not used. Thus, we can guarantee that the function gotten is on the interval [0,1]. It is well known that if a function is defined on the interval [0,1], this function will be a PDF. ☐

The location-scale form of this distribution is given by the following form: suppose that *Z* is distributed as $\mathrm{ABEP}(\alpha_{1},\alpha_{0},\delta_{1},\delta_{0},k_{1},k_{0},\varepsilon )$. Then, the random variable X=μ+σZ will have ABEP distribution with the following density function:

(12) $$g\left(x\right)=\left\{\begin{array}{cc} g_{1}\left(x\right)=\frac{\alpha_{1}}{2\sigma {\left[k_{1}\left(1-\varepsilon \right)\right]}^{\delta_{1}+1}\Gamma \left(\frac{\delta_{1}+1}{\alpha_{1}}\right)}{\left(-\frac{x-\mu }{\sigma }\right)}^{\delta_{1}}\exp\left\{-{\left[\frac{-(x-\mu )}{\sigma k_{1}\left(1-\varepsilon \right)}\right]}^{\alpha_{1}}\right\}, & x<\mu ,\\ g_{0}\left(x\right)=\frac{\alpha_{0}}{2\sigma {\left[k_{0}\left(1+\varepsilon \right)\right]}^{\delta_{0}+1}\Gamma \left(\frac{\delta_{0}+1}{\alpha_{0}}\right)}{\left(\frac{x-\mu }{\sigma }\right)}^{\delta_{0}}\exp\left\{-{\left[\frac{x-\mu }{\sigma k_{0}\left(1+\varepsilon \right)}\right]}^{\alpha_{0}}\right\}, & x\geq \mu , \end{array}\right.$$

where μ∈R and σ>0 are the location and the scale parameters, respectively. Here, we denote the distribution of *X* by $\mathrm{ABEP}(\mu ,\sigma ,\alpha_{1},\alpha_{0},\delta_{1},\delta_{0},k_{1},k_{0},\varepsilon )$ and write $X∼\mathrm{ABEP}(\mu ,\sigma ,\alpha_{1},\alpha_{0},\delta_{1},\delta_{0},k_{1},k_{0},\varepsilon )$. Note that the role of parameters has been given in Theorem 1.

### 2.1. Properties of ABEP Distribution

#### 2.1.1. Mode of a Kernel Function in ABEP

The mode of function in Equation (12) is examined. It is obvious that this function is a reflected function in Equation (3) that comes from the reparameterized gamma function with the power parameter α. Thus, examining the mode of the positive side of Equation (3) means that the negative side of Equation (3) is also examined. Now, it is examined whether or not there is one root of the following function:

(13) $$h\left(t\right)=t^{\delta_{0}}\exp\left\{-t^{\alpha_{0}}\right\},\;\;\;\;t>0,\;\delta_{0}>0,\;\alpha_{0}>0.$$

Here, we will give comments about getting the root of this function: NC can be ignored because NC produces a function at interval [0,1]. It does not affect the modes of function. At the same way, the location parameter μ can be ignored because the location shows where the function in Equation (12) is located. The scale σ, its variants $k_{0}$ or $k_{1}$ and ε parameters change the rescaling of the function in Equation (12).

The root of derivative of the function in Equation (13) with respect to *t* is $\exp\{\alpha_{0}^{-1}\log\left(\alpha_{0}^{-1}\delta_{0}\right)\}$. For t=0, h(t)=0 is the obvious root that does not lead to modality. Thus, there is only one root of function in Equation (13), that is, there is one mode of function of generalized gamma on the positive side. Since it is reflected on the negative side of the real line, the function has a mode at the negative side of the real line. In total, this function in Equation (12) has two modes at the real line. Note that it is not necessary to use a second derivative test because maximization of a function is equivalent to the negative version of that function. Detecting the root is enough for having modality.

#### 2.1.2. Cumulative Distribution Function of ABEP Distribution

Let $X∼\mathrm{ABEP}(\mu ,\sigma ,\alpha_{1},\alpha_{0},\delta_{1},\delta_{0},k_{1},k_{0},\varepsilon )$. Let *G* be CDF of PDF *g*. Then, CDF of the random variable *X* is:

(14) $$G\left(x\right)=\left\{\begin{array}{cc} G_{1}\left(x\right)=\frac{1}{2\Gamma \left(\frac{\delta_{1}+1}{\alpha_{1}}\right)}\Gamma \left(\frac{\delta_{1}+1}{\alpha_{1}},{\left(\frac{-(x-\mu )}{k_{1}\sigma \left(1-\varepsilon \right)}\right)}^{\alpha_{1}}\right), & x<\mu ,\\ G_{0}\left(x\right)=\frac{1+\varepsilon }{2}+\frac{1}{2\Gamma \left(\frac{\delta_{0}+1}{\alpha_{0}}\right)}\gamma \left(\frac{\delta_{0}+1}{\alpha_{0}},{\left(\frac{x-\mu }{k_{0}\sigma \left(1+\varepsilon \right)}\right)}^{\alpha_{0}}\right), & x\geq \mu , \end{array}\right.$$

where γ and Γ are the lower and upper incomplete gamma functions, respectively.

#### 2.1.3. *r*th Moment of Random Variable *X* Distributed as ABEP

Let $X∼\mathrm{ABEP}(\mu =0,\sigma =1,\alpha_{1},\alpha_{0},\delta_{1},\delta_{0},k_{1},k_{0},\varepsilon )$. The *r*th, r≥0, non-central moment is given by

(15) $$E\left(X^{r}\right)=\frac{{\left[k_{1}\left(1-\varepsilon \right)\right]}^{r}\Gamma \left(\frac{\delta_{1}+r+1}{\alpha_{1}}\right)}{2\Gamma \left(\frac{\delta_{1}+1}{\alpha_{1}}\right)}+\frac{{\left[k_{0}\left(1+\varepsilon \right)\right]}^{r}\Gamma \left(\frac{\delta_{0}+r+1}{\alpha_{0}}\right)}{2\Gamma \left(\frac{\delta_{0}+1}{\alpha_{0}}\right)}.$$

One can get the results via Equations (6) and (9). Since $E(X^{r})$ is finite for finite values of parameters $\alpha_{1},\alpha_{0},\delta_{1},\delta_{0},k_{1},k_{0}$ and when the extremely big values of parameters $\alpha_{1},\alpha_{0},\delta_{1},\delta_{0},k_{1},k_{0}$ and *r* are not taken, the ABEP distribution can produce finite values for the estimates of parameters because finiteness of moments guarantees having a finite value of function [35]. Note that the domain of skewness parameter ε is the interval (-1,1).

#### 2.1.4. Moment Generating Function for Random Variable *X* Distributed as ABEP

Let $X∼\mathrm{ABEP}(\mu =0,\sigma =1,\alpha_{1},\alpha_{0},\delta_{1},\delta_{0},k_{1},k_{0},\varepsilon )$. The moment generating function of the random variable *X* is:

(16) $$E\left[\exp\left(tX\right)\right]=\sum_{m=0}^{\infty }\left[\frac{t^{m}{\left[k_{1}\left(1-\varepsilon \right)\right]}^{m}\Gamma \left(\frac{\delta_{1}+m+1}{\alpha_{1}}\right)}{2\Gamma \left(\frac{\delta_{1}+1}{\alpha_{1}}\right)m!}+\frac{t^{m}{\left[k_{0}\left(1+\varepsilon \right)\right]}^{m}\Gamma \left(\frac{\delta_{0}+m+1}{\alpha_{0}}\right)}{2\Gamma \left(\frac{\delta_{0}+1}{\alpha_{0}}\right)m!}\right],$$

where t∈R and m∈N. In order to calculate the integral E[exp(tX)], the Taylor expansion at x=0 of the function $\exp\left(tx\right)=\sum_{m=0}^{\infty }\frac{{\left(tx\right)}^{m}}{m!}$ must be gotten. After some straightforward calculation for the integral E[exp(tX)] via using Equations (6) and (9), the result of integral can be obtained.

#### 2.1.5. PDFs for Different Values of Parameters in ABEP

**Figure 1:**
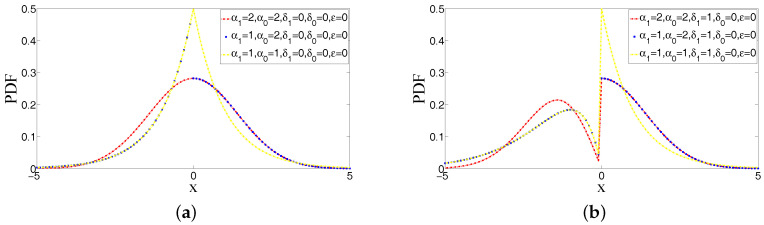
Examples of probability density functions (PDFs) of asymmetric bimodal exponential power (ABEP) distribution for the different values of parameters (μ=0,σ=1): Unimodality, bimodality, half of Laplace and half of normal. (**a**) Unimodal densities due to $\delta_{1}=\delta_{0}=0$, examples for normal and Laplace and their half forms due to $\alpha_{1}>0$ and $\alpha_{0}>0$; (**b**) bimodal densities due to $\delta_{1}>0$, right of density is normal and Laplace due to $\delta_{0}=0$ and $\alpha_{0}$.

**Figure 2:**
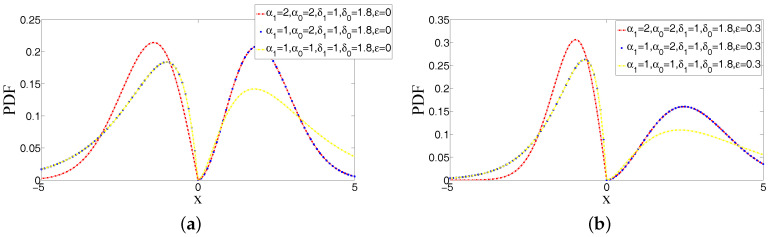
Examples of PDFs of ABEP distribution for the different values of parameters (μ=0,σ=1): Bimodality. (**a**) Bimodal densities constructed via $\delta_{1}>0$, $\delta_{0}>0$, $\alpha_{1}>0$ and $\alpha_{0}>0$; (**b**) bimodal densities via $\delta_{1}>0$, $\delta_{0}>0$, $\alpha_{1}>0$ and $\alpha_{0}>0$ with skewed form: the left and right sides of location have unequal probabilities due to ε.

Figure 1 and Figure 2 illustrate the examples of PDFs of ABEP distribution for some values of parameters that give all possible shapes of function. As can be seen from these figures, the shape of peakedness, bimodality and asymmetry can be controlled at the same time via parameters in ABEP. When different values of parameters $\alpha_{1}$, $\alpha_{0}$ and $\delta_{0}$, $\delta_{1}$ are chosen, a different shape of peakedness and a bimodality with different heights around location parameter μ are obtained, respectively. The skewness parameter ε makes an asymmetry around parameter μ.

#### 2.1.6. Tail Behaviour Property of ABEP

Tail behaviour or heavy tailedness of a distribution is examined by means of definitions given below [36]:

**Definition** **1.** *Let $\bar{G}\left(x\right)$ be 1-G(x). If $\lim_{x\to +\infty }\exp\left(\lambda x\right)\bar{G}\left(x\right)=\infty$ for all λ>0, then G(x) is a heavy-tailed distribution.*

From Equation (14), the positive part of CDF includes the lower incomplete gamma function γ. The function γ(a,b) is examined to get the limit in Definition 1. For b>a, this function goes to zero. Then, $\lim_{x\to +\infty }\exp\left(\lambda x\right)G\left(x\right)$ can go to zero when *b* is more bigger than *a*. Otherwise, this limit is infinite. If $\lim_{x\to +\infty }\exp\left(\lambda x\right)G\left(x\right)\to 0$, then $\lim_{x\to +\infty }\exp\left(\lambda x\right)\bar{G}\left(x\right)\to \infty$ for b>a in γ function.

$\lim_{x\to +\infty }\exp\left(\lambda x\right)\bar{G}\left(x\right)$ is undefined for a case a≥b. It is seen that when *b* as a variable *x* of the function γ has big values, that is, an outlier is included by data, the heavy-tailedness property of ABEP can be obtained. For a≥b, there is already a tendency to get small values of variable *x* in γ function in Equation (14), which does not correspond to an outlier in the data set when it is compared with case b>a in γ function. Thus, having an undefined value for $\lim_{x\to +\infty }\exp\left(\lambda x\right)\bar{G}\left(x\right)$ is not a problem in order to test the heavy-tailedness property of function *G* via Definition 1.

**Definition** **2.** *Suppose that random variable X has a PDF g defined on [0,∞). If E[exp(tX)]=∞, for all t, then g is a heavy-tailed distribution.*

Note that the generalized gamma distribution is reflected to a negative axis or x<μ. The tail behaviour at x>μ or x<μ has the same role. Then, Definition 2 can be used for ABEP.

From Equation (16), E[exp(tX)]=∞ is satisfied due to *m* in summation in Equation (16) of ABEP distribution because *m* goes to infinity and Γ function gives infinity for big values of *m*. Then, ABEP is a heavy-tailed distribution.

A comment for heavy-tailedness from the results of Definitions 1 and 2 is given: the skewness parameter ε and also shape parameters $\alpha_{1},\alpha_{0},\delta_{1},\delta_{0}$ work together in order to get a heavy-tailed function because they are responsible for changing the shape of function.

### 2.2. Special Cases, Related Distributions and Flexibility of ABEP

When we want to make a comparison among distributions in [28,29] and ABEP, the ordered form from lowest to highest for capacity on modelling frequency is to be [28,29] and ABEP distribution. For this reason, ABEP distribution is defined by using the generalized gamma distribution. The obtained distribution has five parameters. Thus, ABEP distribution will have some properties: when $\alpha_{1}=1$ and $\alpha_{0}=2$, the left side of the location is half of Laplace distribution and the right side of location is half of normal distribution for ε=0 and $\delta_{1}=\delta_{0}=0$. For values of $\alpha_{1}=2$ and $\alpha_{0}=1$, the obtained function will be vice versa from the previous case. For these situations, when ε≠0, ABEP will be ε-skew form of half from Laplace and normal distributions. It is easily seen that ABEP distribution can be a combination of Laplace and normal distributions for values of peakedness parameters $\alpha_{1}$ and $\alpha_{0}$ of distribution in ε-skew form. The nuisance parameters $k_{1}$ and $k_{0}$ are added to have the same form of normal and Laplace distributions and also ABEP can have the same framework with algebraic and exponential power distributions in references at items given below. The location-scale form is also provided for ABEP in Equation (12). The parameters $\alpha_{1},\delta_{1}$ and $\alpha_{0},\delta_{0}$ determine the overall shape of function for x<μ and x≥μ, respectively. Tails at negative and positive sides of the real line can be platykurtic ($\alpha_{1},\alpha_{0}\to \infty$) and leptokurtic ($\alpha_{1},\alpha_{0}\to 0$). Note that the random variable *T* in the variable transformation $Z=Y^{1/\alpha }T$ has the same role with ε-skew approach in [33,37,38]. Thus, ABEP is a general scheme for distributions in the class of algebraic and exponential functions. The special cases, the related distributions and the flexibility of ABEP distribution are given in the following items:

1. When $\alpha_{1}=\alpha_{0}=\alpha >0$, ABEP distribution drops to the kernel of distribution in [29].
2. If $\delta_{0}=\delta_{1}=\delta >0$, the density function has two modes (bimodal case) with the same height. If $\delta_{0}=\delta_{1}=0$, the distribution is a unimodal.
3. When ε=0, the distribution is the symmetric with two different modes.
4. When $\alpha_{1}=\alpha_{0}=2$, $\delta_{1}=\delta_{0}=0$, $k_{1}=k_{0}=\sqrt{2}$ and ε=0, ABEP is the standard normal distribution.
5. When $\alpha_{1}=\alpha_{0}=1$, $\delta_{1}=\delta_{0}=0$, $k_{1}=k_{0}=1$ and ε=0, ABEP is the standard Laplace distribution.
6. When $\alpha_{1}=\alpha_{0}=\alpha >0,\delta_{1}=\delta_{0}=\delta >0$, ε=0 and $k_{0}=k_{1}=1$, the distribution is BEP in [28].
7. When $\alpha_{1}=\alpha_{0}=2$ and $\delta_{1}=\delta_{0}=\delta >0$, ABEP distribution is used to model the bimodality with ε-skew asymmetry in its modes on the left and right sides of location μ∈R, which is a similar manner to [9].
8. When $\delta_{1}=\delta_{0}=k-1$, $\alpha_{1}=\alpha_{0}=1$, the ABEP distribution is the same manner as ε-skew gamma distribution in [5].
9. When $\alpha_{1}=\alpha_{0}=2,\delta_{1}=\delta_{0}=0$ and $k_{1}=k_{0}=\sqrt{2}$, the distribution becomes the ε-skew normal distribution in [33]. Note that the random variable *T* is different from distribution in [33].
10. When $\alpha_{1}=\alpha_{0}=1,\delta_{1}=\delta_{0}=0$ and $k_{1}=k_{0}=1$, the distribution becomes the ε-skew Laplace distribution in [37].
11. When $\alpha_{1}=\alpha_{0}=\alpha >0$, $\delta_{1}=\delta_{0}=0$, $k_{1}=k_{0}=1$ and ε=0, ABEP is the generalized normal or Gaussian (exponential power, abbreviated as EP) distribution in [39].
12. When $\delta_{1}=\delta_{0}=0$, ε=0, $\alpha_{1}=\alpha_{0}=2/b$, b∈(0,2] in [40], $\delta_{1}=\delta_{0}=0$, $\alpha_{1}=\alpha_{0}=\alpha >0$, $\kappa_{1}=1-\varepsilon$, $\kappa_{0}=1+\varepsilon$, ε∈(-1,1) in [41], $\delta_{1}=\delta_{0}=0$, a rescaling via convex combination in [42], $\delta_{1}=\delta_{0}=0$, a skewed form via a rescaling in [43,44] and $\delta_{1}=\delta_{0}=0$, ε-skew form in [45], the skewed EP and the symmetric EP distributions are equivalent to distributions in [40,41,42,43,44,45]. The asymmetric EP distributions based on different sense of skewed form of symmetric EP distribution are in [41,42,43,44]. The special functions in Equations (6) and (9) can be used to get the same kernel of EP with recalculated NC in [40,41,42,43,44,45].
13. The ε-skew EP distribution in [38,46] is the special case of this family for $\delta_{0}=\delta_{1}=0$ and $k_{1}=k_{0}=\sqrt{2}$.
14. The kernel of EP distribution without bimodality in [47,48,49] is in the same framework as the special case of ABEP when $k_{1}=k_{0}=k>0$, $\delta_{1}=\delta_{0}=0$ and $\alpha_{1}=\alpha_{0}=\alpha >0$.
15. When the variable transformation $z=y^{1/\alpha }$ on function in Equation (1) is done,
  (17) $$f\left(z\right)=\frac{\alpha }{\Gamma (\frac{\delta +1}{\alpha })}z^{\delta }\exp\left\{-z^{\alpha }\right\},\;\;\;\;z>0,\;\delta >0,\;\alpha >0$$
  is obtained. This is also called as generalized gamma (GG) distribution. The Pearson type III and V, Erlang, exponential, Weibull, Pareto, Levy, Rayleigh, Nakagami, Frechet, Helmert, Maxwell–Boltzmann and four-parameter exponential gamma as algebraic and exponential functions are members of function in Equation (17) [31,50,51,52] and references therein.

The first developer of EP is in [47] via solving the differential equation as a different sense from GG in Equation (17). The EP as generalized error distribution was proposed by [48]. In ABEP distribution, there are parameters for modelling x<μ and x≥μ. Thus, the bimodality can be produced (see also Section 2.1.1) and the role of parameters that creates bimodality due to reflection approach in Equation (2) of GG function can be observed easily.

## 3. Maximum Likelihood Estimations for Parameters of ABEP Distribution

Let $x_{1},x_{2},...,x_{n}$ be a random sample of size *n* from an ABEP distributed population. The unknown parameters $\mu ,\sigma ,\alpha_{1},\alpha_{0},\delta_{1},\delta_{0}$ and ε will be estimated by an ML estimation method [35]. Here, the parameters $k_{1}$ and $k_{0}$ are nuisance parameters. The log-likelihood log(L) function is:

(18) $$\begin{array}{cc} \log[L(x;\theta )]= & n_{1}\left[\log\left(\alpha_{1}\right)-\log\left(2\sigma {\left[k_{1}\left(1-\varepsilon \right)\right]}^{\delta_{1}+1}\right)-\log\left(\Gamma \left(\frac{\delta_{1}+1}{\alpha_{1}}\right)\right)\right]\\ \; & +\delta_{1}\sum_{i=1}^{n_{1}}\log\left(\frac{-(x_{i}-\mu )}{\sigma }\right)-\sum_{i=1}^{n_{1}}{\left(\frac{-(x_{i}-\mu )}{\sigma [k_{1}\left(1-\varepsilon \right)]}\right)}^{\alpha_{1}}\\ \; & +n_{0}\left[\log\left(\alpha_{0}\right)-\log\left(2\sigma {\left[k_{0}\left(1+\varepsilon \right)\right]}^{\delta_{0}+1}\right)-\log\left(\Gamma \left(\frac{\delta_{0}+1}{\alpha_{0}}\right)\right)\right]\\ \; & +\delta_{0}\sum_{i=1}^{n_{0}}\log\left(\frac{x_{i}-\mu }{\sigma }\right)-\sum_{i=1}^{n_{0}}{\left(\frac{x_{i}-\mu }{\sigma [k_{0}\left(1+\varepsilon \right)]}\right)}^{\alpha_{0}}, \end{array}$$

where $n_{0}$ is the number of non-negative observations and $n_{1}$ is the number of negative observations. $\hat{\theta }=\left(\hat{\mu },\hat{\sigma },{\hat{\alpha }}_{1},{\hat{\alpha }}_{0},{\hat{\delta }}_{1},{\hat{\delta }}_{0},\hat{\varepsilon }\right)$ are ML estimators of parameter vector $\theta =(\mu ,\sigma ,\alpha_{1},\alpha_{0},\delta_{1},\delta_{0},\varepsilon )$.

The second derivative test can be used to determine whether or not the log(L) function in Equation (18) has the maximum value. However, since PDF has seven parameters $\mu ,\sigma ,\alpha_{1},\alpha_{0},\delta_{1},\delta_{0}$ and ε, it is not easy to get a Hessian matrix because of two cases and seven parameters in ABEP. One can get it via using the mathematical software programs, which are Maple 18.00 (Maplesoft, Waterloo, ON, Canada) or Mathematica 9.0.1.0 (Wolfram Research, Champaign, IL, USA). It is also noted that ABEP can have a discontinuity point at x=μ for some values of parameters. There can be a solution to overcome this problem if we focus on improving the modelling capacity of PDF having more parameters, which help us to increase flexibility of the function. Thus, the efficiency for ML estimators of the parameters μ and σ is increased. A solution in an indirect way for this problem is that one can use GOFT statistics, such as KS, CVM and AD to see the distances between expected and empirical cumulative distributions. It is well known that the smaller values of the GOFT statistics mean that more fitting performance accomplished by the function. In the computation process, optimization of nonlinear function in Equation (18) is conducted via hybrid genetic algorithm (HGA) in MATLAB 2016a (MathWorks, Natick, MA, USA). In HGA, intervals for parameters that will optimize the log(L) function in Equation (18) are used. The intervals for $\mu ,\sigma ,\alpha_{1},\alpha_{0},\delta_{1},\delta_{0}$ and ε are [-5,5],[0,5],[0,10],[0,10],[0,10],[0,10] and (-1,1), which is a domain of skewness parameter ε. $k_{1}$ and $k_{0},$ as nuisance parameters are taken to be $\alpha_{1}$ and $\alpha_{0}$. This form is appropriate to have the same form of normal and Laplace. Let us remind that ABEP is a generalized normal or Laplace distribution. Thus, $k_{1}$ and $k_{0}$ are nuisance parameters.

The Fisher information matrix for parameters μ and σ from ABEP is given by matrix *F* in the following form:

(19) $$F\left(\theta \right)=\left[\begin{array}{cc} E_{1}\left[\frac{\partial^{2}\log\left[f\left(x;\mu ,\sigma \right)\right]}{\partial \mu^{2}}\right]+E_{0}\left[\frac{\partial^{2}\log\left[f\left(x;\mu ,\sigma \right)\right]}{\partial \mu^{2}}\right] & E_{1}\left[\frac{\partial^{2}\log\left[f\left(x;\mu ,\sigma \right)\right]}{\partial \mu \partial \sigma }\right]+E_{0}\left[\frac{\partial^{2}\log\left[f\left(x;\mu ,\sigma \right)\right]}{\partial \mu \partial \sigma }\right]\\ E_{1}\left[\frac{\partial^{2}\log\left[f\left(x;\mu ,\sigma \right)\right]}{\partial \mu \partial \sigma }\right]+E_{0}\left[\frac{\partial^{2}\log\left[f\left(x;\mu ,\sigma \right)\right]}{\partial \mu \partial \sigma }\right] & E_{1}\left[\frac{\partial^{2}\log\left[f\left(x;\mu ,\sigma \right)\right]}{\partial \sigma^{2}}\right]+E_{0}\left[\frac{\partial^{2}\log\left[f\left(x;\mu ,\sigma \right)\right]}{\partial \sigma^{2}}\right] \end{array}\right].$$

The Cramér–Rao lower bounds (RCLBs) for ML estimators of parameters are given. The Monte Carlo numerical integration is used to compute the integrals in Fisher information in Equation (19) for RS, ESC and ASL distributions.

Equations (6) and (9) are used to calculate the integrals in matrix *F*. Due to the analytical expression of PDF in Equation (12), off-diagonal elements of matrix *F* are non-zero. Here, shape $\alpha_{1}$, $\alpha_{0}$, bimodality $\delta_{1}$, $\delta_{0}$, skewness ε and nuisance $k_{1}$, $k_{0}$ parameters make a covariance structure between location μ and scale σ parameters. From this result, covariance structure on ML estimators of other parameters can be seen. Since it is possible to obtain the covariance among ML estimators, the Fisher information matrix is obtained only for the ML estimators of two parameters μ and σ. If there can be a covariance among ML estimators, the inverse of matrix *F* cannot be obtained except the generalized inverse. Note that getting matrix *F* for μ and σ from ABEP is tractable for the calculation of integration of Fisher information. Using the generalized inverse cannot be preferable due to a loss of information in an inverse of a matrix. Loss of information occurs because the multiplication of generalized inverse of matrix *F* and *F*, that is, $F^{-}F$, does not give an identity matrix [53]. When $\alpha_{1}=\alpha_{0}=\alpha$, $\delta_{1}=\delta_{0}=\delta$, ε=0 and $k_{1}=k_{0}=k$, $E_{1}\left[\frac{\partial^{2}\log\left[f\left(x;\mu ,\sigma \right)\right]}{\partial \mu \partial \sigma }\right]+E_{0}\left[\frac{\partial^{2}\log\left[f\left(x;\mu ,\sigma \right)\right]}{\partial \mu \partial \sigma }\right]=0$, that is, the covariance between ML estimators of μ and σ from ABEP is zero:

(20) $$\begin{array}{ccc} E_{1}\left[\frac{\partial^{2}\log\left[f\left(x;\mu ,\sigma \right)\right]}{\partial \mu^{2}}\right] & = & \frac{\delta_{1}\Gamma \left(\frac{\delta_{1}-1}{\alpha_{1}}\right)+\alpha_{1}\left(\alpha_{1}-1\right)\Gamma (1-\frac{1-\delta_{1})}{\alpha_{1}}}{2{\left[\sigma k_{1}\left(1-\varepsilon \right)\right]}^{2}\Gamma \left(\frac{\delta_{1}+1}{\alpha_{1}}\right)}, \end{array}$$

(21) $$\begin{array}{ccc} E_{0}\left[\frac{\partial^{2}\log\left[f\left(x;\mu ,\sigma \right)\right]}{\partial \mu^{2}}\right] & = & \frac{\delta_{0}\Gamma \left(\frac{\delta_{0}-1}{\alpha_{0}}\right)+\alpha_{0}\left(\alpha_{0}-1\right)\Gamma (1-\frac{1-\delta_{0})}{\alpha_{0}}}{2{\left[\sigma k_{0}\left(1+\varepsilon \right)\right]}^{2}\Gamma \left(\frac{\delta_{0}+1}{\alpha_{0}}\right)}, \end{array}$$

(22) $$\begin{array}{ccc} E_{1}\left[\frac{\partial^{2}\log\left[f\left(x;\mu ,\sigma \right)\right]}{\partial \mu \partial \sigma }\right] & = & \frac{-\alpha_{1}^{2}\Gamma \left(1+\delta_{1}/\alpha_{1}\right)}{2k_{1}\left(1-\varepsilon \right)\sigma^{2}\Gamma \left(\frac{\delta_{1}+1}{\alpha_{1}}\right)}, \end{array}$$

(23) $$\begin{array}{ccc} E_{0}\left[\frac{\partial^{2}\log\left[f\left(x;\mu ,\sigma \right)\right]}{\partial \mu \partial \sigma }\right] & = & \frac{\alpha_{0}^{2}\Gamma \left(1+\delta_{0}/\alpha_{0}\right)}{2k_{0}\left(1+\varepsilon \right)\sigma^{2}\Gamma \left(\frac{\delta_{0}+1}{\alpha_{0}}\right)}, \end{array}$$

(24) $$\begin{array}{ccc} E_{1}\left[\frac{\partial^{2}\log\left[f\left(x;\mu ,\sigma \right)\right]}{\partial \sigma^{2}}\right] & = & \frac{1}{2\sigma^{2}}\left[-1-\delta_{1}+\frac{\alpha_{1}\left(\alpha_{1}+1\right)\Gamma \left(1+\frac{\delta_{1}+1}{\alpha_{1}}\right)}{\Gamma (\frac{\delta_{1}+1}{\alpha_{1}})}\right], \end{array}$$

(25) $$\begin{array}{ccc} E_{0}\left[\frac{\partial^{2}\log\left[f\left(x;\mu ,\sigma \right)\right]}{\partial \sigma^{2}}\right] & = & \frac{1}{2\sigma^{2}}\left[-1-\delta_{0}+\frac{\alpha_{0}\left(\alpha_{0}+1\right)\Gamma \left(1+\frac{\delta_{0}+1}{\alpha_{0}}\right)}{\Gamma (\frac{\delta_{0}+1}{\alpha_{0}})}\right]. \end{array}$$

Some of the regularity conditions [35] are as follows:

1. det[F(μ,σ)]<∞ and
2. $|\frac{\partial^{3}}{\partial \theta^{3}}\log f\left(x;\theta \right)|\leq M\left(x\right).$ Then, E[M(X)]<∞.

One can verify that the conditions can be satisfied by using the mathematical software programs, which are Maple 18.00 (Maplesoft, Waterloo, ON, Canada) or Mathematica 9.0.1.0 (Wolfram Research, Champaign, IL, USA). Here, it is possible to get M(X) as $X^{r}$ in Equation (15). Then, the condition 2 is satisfied. The other regularity conditions are already satisfied obviously. Since the ABEP distribution satisfies these two conditions,

(26) $$\sqrt{n}\left(\left[\begin{array}{c} \hat{\mu }\\ \hat{\sigma } \end{array}\right]-\left[\begin{array}{c} \mu \\ \sigma \end{array}\right]\right)\overset{D}{⟶}N\left(0,{\left[F\left(\mu ,\sigma \right)\right]}^{-1}\right),$$

that is, $\sqrt{n}\left(\left[\begin{array}{c} \hat{\mu }\\ \hat{\sigma } \end{array}\right]-\left[\begin{array}{c} \mu \\ \sigma \end{array}\right]\right)$ is asymptotically normal with mean zero vector and covariance matrix ${\left[F\left(\mu ,\sigma \right)\right]}^{-1},$ and $\hat{\mu }$, $\hat{\sigma }$ are asymptotically efficient and asymptotic normally distributed [35].

## 4. Simulation Study

In this section, a brief simulation study to verify the behaviour of the ML estimators in finite samples is presented. The data were drawn based on the stochastic representation given in Appendix A. The arbitrary values for the parameters are chosen. The probable handling for the values of parameters is considered such that the different combination for the values of $\alpha_{1}$, $\alpha_{0}$, $\delta_{1}$ and $\delta_{0}$ can be observable. Thus, an asymmetry and a bimodality can be constructed. Table 1 has the chosen values for the parameters. Three sample sizes are utilized: 100, 200 and 500. Based on 1000 replicates for each sample size, the average bias and the root of the mean squared error (RMSE) are computed and they are given in Table 1. HGA is used to optimize the log(L) function in Equation (18) according to the parameters $\mu ,\sigma ,\alpha_{1},\alpha_{0},\delta_{1},\delta_{0}$ and ε. In the computation process of HGA, the intervals for $\mu ,\sigma ,\alpha_{1},\alpha_{0},\delta_{1},\delta_{0}$ and ε are [-5,5],[0,5],[0,10],[0,10],[0,10],[0,10] and (-1,1).

In general, it is observed from the results of simulation that the bias can be acceptable and it can decrease when the sample size increases. When the sample size *n* increases, the values of $\hat{\mathrm{RMSE}}$ go to zero because the function is represented well by the artificial data, which is an expected result.

## 5. Real Data Illustration

In this section, the modelling capability of ABEP is shown by applying it to two data sets from microarray [54]. The real data sets from the web site in [54] are given in Table A1 and Table A2 in Appendix B. The analysis of proteins in cancer cells is important. The efficient estimates of location and scale parameters for these proteins have a crucial role in medical care. For this reason, we prefer to focus on these data sets that have different shapes of peakedness, bimodality and asymmetry.

In the second step, the distributions are considered to model these data sets. In the estimation process, we use the maximum likelihood method together with GOFT statistics, mostly prominent ones that are KS, CVM and AD (robust one) distances to test the fitting capability of distributions [55]. When the estimates of parameters are computed, we can examine via GOFT statistics which of the five PDFs is the best fit on data.

The bimodal extended generalized gamma (BEGG) [29], the Rathie-Swamee (RS) (RS is also known to be a modified version of generalized logistic) [11,12,13], the exponentiated sinh Cauchy (ESC) [10] and the alpha-skew Laplace (ASL) [24] distributions are used to fit the data and make a comparison between them and ABEP. There are many different distributions that have been proposed; however, using an explicit expression for CDF would be preferred to fit the data. For this reason, the distributions having an explicit expression for their CDFs are used. Thus, GOFTs can be used without including the numerical integration methods having computational errors.

Modelling data (or Riemann integration in randomly putting the bin of histograms on the real line) is equivalent to an integration. Thus, the discontinuity at x=μ is not a problem for estimations of parameters. For computation, the HGA is used. HGA also includes the derivative free approach [56] for optimization. Then, the discontinuity point x=μ is not a problem for optimization of log(L) function in Equation (18) according to parameters. At the same time, GOFT statistics are used while performing the computation process.

The algorithm given in Appendix A allows for generating a random variable that is distributed according to a PDF given in Equation (12). Thus, the performance of fitting can be checked via the counted data at the prescribed ranges of domain. However, this procedure is rough when it is compared with GOFTs. It is also beneficial to observe the performance of the random number generation procedure.

The number of replicated sample size *n* is 100,000. Data generated from ABEP, BEGG and ESC distributions are sorted from small to big values for each sample size *n*. After sorting, arithmetic mean of 100,000 artificial data is obtained for n=118. After artificial data are generated from their corresponding PDFs, it is also possible to check the fitting performance of these functions via the artificial data (see Tables 4 and 7). Since ABEP, BEGG and ESC are competitive distributions for fitting data and they have a random number generation procedure, they are preferred to check their similarities with real data. In two examples given in the following subsections, Tables 2 and 5 give the ML estimates of parameters in PDFs and GOFT statistics for these estimated values of parameters. Tables 3 and 6 show the asymptotic variances and covariances of ML estimators for the parameters μ and σ. The Monte Carlo numerical integration method is used while performing the computation process of integration in the Fisher information matrix. Tables 4 and 7 represent the counted data at the prescribed ranges of domain.

### 5.1. Example 1: Modelling Shape of Peakedness, Bimodality and Asymmetry

The data labelled as “Homo sapiens Pig7 (PIG7) mRNA, complete cds Chr.16 [381663, (EW), 5’:AA059047, 3’:AA059031]” from microarray are modelled by ABEP, BEGG, RS, ESC and ASL distributions. Table 2 gives the ML estimates of parameters in these distributions and GOFT statistics for these estimated values of parameters μ, σ, $\alpha_{1}$, $\alpha_{0}$, $\delta_{1}$, $\delta_{0}$ and ε. Table 3 shows the asymptotic variances and covariances of ML estimators for the parameters μ and σ. Table 4 represents the counted data at the ranges [−10, −0.3, −0.1, 0, 0.1, 0.3, 10].

### 5.2. Example 2: Modelling Shape of Peakedness, Bimodality and Asymmetry

The data from microarray labelled as “SID 377353, ESTs [5’:, 3’:AA055048]” are modelled by ABEP, BEGG, RS, ESC and ASL distributions. Table 5 gives the ML estimates of parameters in these distributions and GOFT statistics for these estimated values of parameters μ, σ, $\alpha_{1}$, $\alpha_{0}$, $\delta_{1}$, $\delta_{0}$ and ε. Table 6 shows the asymptotic variances and covariances of ML estimators for the parameters μ and σ. Table 7 represents the counted data at the ranges [−10, −0.4, −0.2, 0, 0.2, 0.4, 10].

### 5.3. Comments on the Results of Examples 1 and 2

**Figure 3:**
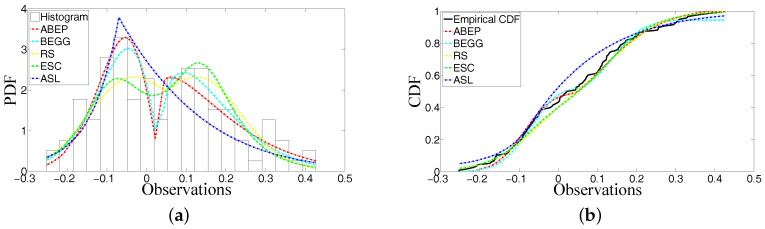
Probability density function (PDF) and Cumulative density function (CDF) for the considered distributions. (**a**) PDF of ABEP (Asymmetric bimodal exponential power), Bimodal extended generalized gamma (BEGG), Rathie-Swamee (RS), exponentiated sinh Cauchy (ESC) and alpha-skew Laplace (ASL) distributions for the estimates of their parameters; (**b**) CDF of ABEP, BEGG, RS, ESC and ASL distributions for the estimates of their parameters.

**Figure 4:**
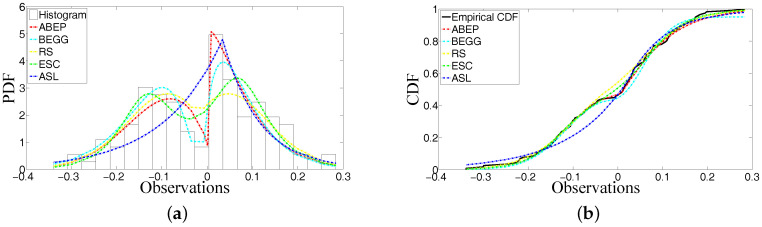
PDF and CDF for the considered distributions. (**a**) PDF of ABEP, BEGG, RS, ESC and ASL distributions for the estimates of their parameters; (**b**) CDF of ABEP, BEGG, RS, ESC and ASL distributions for the estimates of their parameters.

For both of the examples, Figure 3a and Figure 4a show that ABEP fits better than the other distributions. In particular, the modalities around location have been modelled as the different modes of heights. The shape of peakedness can be modelled as well. The right side of the location is especially modelled very well by ABEP in Example 2. The asymmetry illustrated in Example 1 has been modelled.

The histograms of data in Example 2 do not show an asymmetry and the ML estimate of skewness parameter is very near to zero because, as it is seen from Figure 4a, the histograms do not have an asymmetry when they are compared with histograms in Figure 3a. The unequally distributed histograms around location in Figure 3a can show that there is an asymmetry in the data set.

For both of the examples, Table 2 and Table 5 give the ML estimates of parameters of distributions and GOFT statistics of fitted densities. ABEP distribution has the best fitting on data when we consider the values of KS and CVM statistics. When we look at the fitting performance for all distributions from Figure 3a and Figure 4a, it is seen that ABEP, BEGG and ESC have better fitting performance than RS and ASL. However, when ABEP and ESC are compared, it is observed that two parameters λ and β of ESC are not enough to get the precise fitting on data because these parameters work together around the location. In BEGG, there is only one parameter α to control the fitting shape of function on the real line. In ABEP, the role of parameters $\alpha_{1},\alpha_{0},\delta_{1}$ and $\delta_{0}$ around the location is constructed definitely. Thus, more precise estimates for parameters μ and σ can be obtained through these parameters if the data are from many phenomena.

It is well known that the probability value (*p*-value) of a test statistic depends on the fitted density. For this reason, more efficient density must be preferred before getting the *p*-value of a test statistic from corresponding density. Then, the potential problem that can occur in future from phenomena can be refrained. The estimates of μ from fitted densities of ABEP, BEGG, RS and ESC can be close to each other, but the estimates of μ of ABEP are more precise because ABEP is the best one for fitting on data. Similarly, the estimates of σ of ABEP for both of examples are the best ones.

The random number generation procedure can be conducted in a more precise way for ABEP, BEGG and ESC distributions because ABEP and BEGG have an algorithm of random number generation in Appendix A. The inverse of CDF of ESC distribution [10] can be taken to get the random numbers from ESC. The artificial data generated from ABEP distribution also show that the counted artificial data at ranges can be similar to the counted real data at ranges (see Table 4 and  Table 7). It is noted that the mostly counted data (the numbers 37 and 62 in Examples 1 and 2, respectively) at an interval for real data are constructed by the artificial data generated from ABEP distribution for the prescribed ranges on the real line. The counted artificial data from ABEP represent the counted real data when they are compared with the counted artificial data from BEGG and ESC. Thus, we can infer that the data generation procedure is also successful after we get the precise estimates of parameters in ABEP via collaboration with GOFT statistics.

GOFT statistics in Table 2 and Table 5 show that there can be a numerical error in the computation of special function from CDF of ABEP. The AD for ABEP can have a numerical error from the computation of CDF because CDF of ABEP is a special function. Even if CDF of ABEP depends on special functions that are incomplete gamma functions, the fitting performance of ABEP is the best one due to the fact that all possible parameters (shape, bimodality and skewness) are added into ABEP.

## 6. Conclusion and Discussion

A family for bimodal distribution with two parameters fitting the shape of peakedness  ($\alpha_{1}$ and $\alpha_{0}$), two parameters fitting the height of bimodality ($\delta_{1}$ and $\delta_{0}$) and a parameter fitting the asymmetry (ε) in the data set have been proposed. The unimodal case of this family is obtained when $\delta_{1}=\delta_{0}=0$. The skewness parameter in this family is from the ε-skew approach, which can produce the asymmetry around location. The importance of having these parameters in ABEP for modelling around locations separately has been observed when we make a comparison among ABEP, BEGG, RS and ESC distributions that have explicit expression for CDF. As a result, ABEP can model efficiently the shape of peakedness, the bimodality and the asymmetry at the same time because ABEP has parameters that are responsible for fitting the shape of peakedness, the bimodality and the asymmetry in data when it is compared with BEGG, RS, ESC and ASL distributions.

The well known approach that derives PDFs without consulting the variable transformation technique is applied for the tractable functions in Equations (6)–(11) to propose a new distribution. It is clear that this approach can be applied for other kinds of distributions that are on the negative, positive or real lines. The disadvantage of this approach is that the analytical expression of a function must be tractable to derive a PDF. Equations (6)–(8) are the power version of gamma, lower and upper incomplete gamma functions. The functions in Equations (9)–(11) are transferred to the negative side of the real line through using functions in Equations (6)–(8). They are a new kind of the special functions to calculate the integrals having the kernel of gamma function. One can get distributions via these functions. For example, alpha-skew Laplace [24], alpha–beta skew normal [3], alpha-skew generalized *t* with variable transformation [57,58], symmetric and asymmetric EP [40,41,42,43,44,45] distributions with the recalculated NC can also be obtained by these special functions. The special cases, the related distributions and the flexibility of ABEP are given in the relevant section.

An algorithm for generating artificial data from ABEP is provided. Thus, the similarity between artificial and real data sets has been observed as a rough approach and the performance of optimization for the log(L) function and GOFTs can be supported by this similarity as well. The benefit of GOFTs is depicted when a PDF has more parameters. The best performance on optimizing the log(L) function can also be checked by GOFT statistics. Thus, if CDF of a PDF exists, using GOFTs as an indirect way to check the potential optimization problem(s) is provided when the second derivative test is a problem for getting the Hessian matrix with respect to parameters of log(L) function. HGA is also used to overcome the problems that can occur while performing optimization of log(L) function according to the parameters in ABEP. As a result, performing a cross check between the optimization tool HGA and the GOFT statistics is a beneficial approach to overcome the potential problem(s) from the computation process. Thus, more precise ML estimates for parameters can be gained. When it is considered on overall results from illustrating of PDF and CDF and also artificial data, the GOFT statistics and these results support each other to show the fitting performance of ABEP. The brief simulation study gives the satisfactory results for the bias and RMSE.

RCLBs for ML estimators of parameters μ and σ are obtained. The properties of ABEP are provided and so the heavy-tailedness property of ABEP distribution has been examined. The heavy-tailedness of ABEP from Definitions 1 and 2 are guaranteed when b>a in the γ function. Definitions 1 and 2 imply that ABEP can be a heavy-tailed distribution together with that comment in there.

The entropy-based parameter estimation for ABEP is an ongoing issue in [31,32] to study via the proposed special functions in Equations (6)–(11). We will introduce the information theoretic model selection criteria [59] to fit the models, and the results will be published separately. In the future, a package in a statistical software R from open access will be prepared for ABEP distribution with different estimation methods [31,55], and the information theoretic model selection criteria will be added into this package.

## Figures and Tables

**Table 1:** $\hat{\mathrm{Bias}}$ and $\hat{\mathrm{RMSE}}$ of ML estimates of vector $\hat{\theta }$.

		$\hat{\mathrm{Bias}}\left(\hat{\theta }\right)$	$\hat{\mathrm{RMSE}}\left(\hat{\theta }\right)$		$\hat{\mathrm{Bias}}\left(\hat{\theta }\right)$	$\hat{\mathrm{RMSE}}\left(\hat{\theta }\right)$		$\hat{\mathrm{Bias}}\left(\hat{\theta }\right)$	$\hat{\mathrm{RMSE}}\left(\hat{\theta }\right)$		$\hat{\mathrm{Bias}}\left(\hat{\theta }\right)$	$\hat{\mathrm{RMSE}}\left(\hat{\theta }\right)$
*n* = 100												
μ	0	−0.0216	0.0859	0	−0.0336	0.0890	0	−0.0252	0.0683	0	0.0014	0.1513
σ	1	−0.0387	0.0751	1	0.0686	0.2349	1	−0.0357	0.0800	1	−0.0573	0.1055
$\alpha_{1}$	2	0.0190	0.3529	1.5	0.1024	0.2937	1.5	0.0372	0.2853	2.5	0.0643	0.4281
$\alpha_{0}$	2	0.0909	0.3503	1	0.1333	0.2753	2.5	0.0455	0.4304	1.5	0.0548	0.3327
$\delta_{1}$	1	0.0613	0.4758	1	−0.1541	0.5550	1	−0.0391	0.4067	2	0.0439	0.4664
$\delta_{0}$	2	0.1128	0.3714	1.5	−0.0128	0.3711	2	0.2130	0.6041	1	0.1109	0.3152
ε	0.5	0.0042	0.0433	0.5	0.0088	0.0933	0.5	0.0004	0.0535	0.5	−0.0170	0.0566
*n* = 200												
μ	0	−0.0141	0.0568	0	−0.0174	0.0607	0	−0.0202	0.0490	0	0.0167	0.1221
σ	1	−0.0314	0.0604	1	0.0388	0.2187	1	−0.0280	0.0608	1	−0.0482	0.0958
$\alpha_{1}$	2	0.0057	0.3294	1.5	0.1078	0.2839	1.5	0.0263	0.2609	2.5	0.0746	0.4013
$\alpha_{0}$	2	0.0602	0.3254	1	0.0839	0.2282	2.5	0.0201	0.4164	1.5	0.0274	0.3006
$\delta_{1}$	1	0.0324	0.3793	1	−0.0999	0.5151	1	−0.0572	0.3671	2	0.0768	0.4546
$\delta_{0}$	2	0.0844	0.3394	1.5	0.0053	0.3444	2	0.1578	0.4987	1	0.0726	0.2957
ε	0.5	−0.0007	0.0326	0.5	−0.0010	0.0929	0.5	−0.0006	0.0435	0.5	−0.0163	0.0474
*n* = 500												
μ	0	−0.0062	0.0383	0	−0.0119	0.0400	0	−0.0103	0.0304	0	0.0265	0.0912
σ	1	−0.0203	0.0452	1	0.0301	0.1989	1	−0.0170	0.0450	1	−0.0296	0.0831
$\alpha_{1}$	2	0.0158	0.2804	1.5	0.0977	0.2658	1.5	0.0345	0.2279	2.5	0.0520	0.3548
$\alpha_{0}$	2	0.0532	0.2941	1	0.0509	0.1762	2.5	0.0106	0.3688	1.5	0.0121	0.2445
$\delta_{1}$	1	0.0176	0.3040	1	−0.0904	0.4393	1	−0.0316	0.3367	2	0.1372	0.4239
$\delta_{0}$	2	0.0388	0.3150	1.5	−0.0061	0.3040	2	0.0836	0.3933	1	0.0209	0.2663
ε	0.5	−0.0009	0.0249	0.5	−0.0023	0.0881	0.5	0.0002	0.0387	0.5	−0.0103	0.0382

RMSE: the root of the mean squared error; ML: Maximum likelihood.

**Table 2:** Maximum likelihood (ML) estimates of parameters and Goodness of fit test (GOFT) statistics of fitted densities for microarray data.

	$\hat{\mu }$	$\hat{\sigma }$	${\hat{\alpha }}_{1}$	${\hat{\alpha }}_{0}$	${\hat{\delta }}_{1}$	${\hat{\delta }}_{0}$	$\hat{\varepsilon }$	KS	CVM	AD
ABEP	0.0395	0.1060	1.7322	1.4499	1.2434	0.0505	0.3864	0.0510	0.0662	0.7150
	$\hat{\mu }$	$\hat{\sigma }$	${\hat{\alpha }}_{1}=\hat{\alpha }$	${\hat{\alpha }}_{0}=\hat{\alpha }$	${\hat{\delta }}_{1}$	${\hat{\delta }}_{0}$	$\hat{\varepsilon }$	KS	CVM	AD
BEGG	0.0389	0.0926	1.4880	1.4880	1.0673	0.2657	0.2261	0.0574	0.0850	0.9568
	$\hat{\mu }$	$\hat{\sigma }$	$\hat{a}$	$\hat{b}$	$\hat{p}$			KS	CVM	AD
RS	0.0468	0.2049	1.6278	0.7525	1.1703			0.0865	0.1229	0.8152
	$\hat{\mu }$	$\hat{\sigma }$	$\hat{\lambda }$	$\hat{\beta }$				KS	CVM	AD
ESC	0.0226	0.0725	0.4091	1.1730				0.0737	0.1052	0.7086
	$\hat{\mu }$	$\hat{\sigma }$	$\hat{a}$					KS	CVM	AD
ASL	−0.0700	0.1052	−0.5039					0.1318	0.4449	2.3821

ABEP: Asymmetric bimodal exponential power; BEGG: Bimodal extended generalized gamma; RS: Rathie–Swamee; ESC: Exponentiated sinh Cauchy; ASL: alpha-skew Laplace; KS: Kolmogorov–Smirnov; CVM: Cramér von Mises; AD: Anderson–Darling.

**Table 3:** Asymptotic variances and covariances of ML estimators $\hat{\mu }$ and $\hat{\sigma }$ ($10^{-3}$).

ABEP		BEGG		RS		ESC		ASL	
$\hat{Var}\left(\hat{\mu }\right)$	$\hat{Cov}\left(\hat{\mu },\hat{\sigma }\right)$	$\hat{Var}\left(\hat{\mu }\right)$	$\hat{Cov}\left(\hat{\mu },\hat{\sigma }\right)$	$\hat{Var}\left(\hat{\mu }\right)$	$\hat{Cov}\left(\hat{\mu },\hat{\sigma }\right)$	$\hat{Var}\left(\hat{\mu }\right)$	$\hat{Cov}\left(\hat{\mu },\hat{\sigma }\right)$	$\hat{Var}\left(\hat{\mu }\right)$	$\hat{Cov}\left(\hat{\mu },\hat{\sigma }\right)$
$\hat{Cov}\left(\hat{\mu },\hat{\sigma }\right)$	$\hat{Var}\left(\hat{\sigma }\right)$	$\hat{Cov}\left(\hat{\mu },\hat{\sigma }\right)$	$\hat{Var}\left(\hat{\sigma }\right)$	$\hat{Cov}\left(\hat{\mu },\hat{\sigma }\right)$	$\hat{Var}\left(\hat{\sigma }\right)$	$\hat{Cov}\left(\hat{\mu },\hat{\sigma }\right)$	$\hat{Var}\left(\hat{\sigma }\right)$	$\hat{Cov}\left(\hat{\mu },\hat{\sigma }\right)$	$\hat{Var}\left(\hat{\sigma }\right)$
0.0215	0.0082	0.0073	0.0014	0.6481	0.0375	0.5739	-0.0174	4.365	0.4549
	0.0383		0.0296		0.0615		0.0756		0.0419

**Table 4:** Counted data at ranges [−10, −0.3, −0.1, 0, 0.1, 0.3, 10].

Real Data	0	22	28	22	37	9	0
ABEP	0	17	29	22	38	12	0
BEGG	0	18	28	22	42	8	0
ESC	1	21	25	25	41	5	0

ABEP: Asymmetric bimodal exponential power; BEGG: Bimodal extended generalized gamma; ESC: Exponentiated sinh Cauchy.

**Table 5:** ML estimates of parameters and GOFT statistics of fitted densities for microarray data.

	$\hat{\mu }$	$\hat{\sigma }$	${\hat{\alpha }}_{1}$	${\hat{\alpha }}_{0}$	${\hat{\delta }}_{1}$	${\hat{\delta }}_{0}$	$\hat{\varepsilon }$	KS	CVM	AD
ABEP	0.0070	0.0810	2.1174	1.3610	0.4937	0.0031	−0.0380	0.0392	0.0203	0.2773
	$\hat{\mu }$	$\hat{\sigma }$	${\hat{\alpha }}_{1}=\hat{\alpha }$	${\hat{\alpha }}_{0}=\hat{\alpha }$	${\hat{\delta }}_{1}$	${\hat{\delta }}_{0}$	$\hat{\varepsilon }$	KS	CVM	AD
BEGG	−0.0113	0.0516	1.0770	1.0770	1.7593	0.8923	−0.0048	0.0763	0.0936	0.7397
	$\hat{\mu }$	$\hat{\sigma }$	$\hat{a}$	$\hat{b}$	$\hat{p}$			KS	CVM	AD
RS	−0.0201	0.3848	2.7876	3.9241	0.6641			0.0996	0.1083	0.5158
	$\hat{\mu }$	$\hat{\sigma }$	$\hat{\lambda }$	$\hat{\beta }$				KS	CVM	AD
ESC	−0.0361	0.0561	0.3143	1.1959				0.0630	0.0396	0.2502
	$\hat{\mu }$	$\hat{\sigma }$	$\hat{a}$					KS	CVM	AD
ASL	0.0340	0.0988	0.2357					0.1099	0.2491	1.5098

ABEP: Asymmetric bimodal exponential power; BEGG: Bimodal extended generalized gamma; RS: Rathie–Swamee; ESC: Exponentiated sinh Cauchy; ASL: alpha-skew Laplace; KS: Kolmogorov–Smirnov; CVM: Cramér von Mises; AD: Anderson–Darling.

**Table 6:** Asymptotic variances and covariances of ML estimators $\hat{\mu }$ and $\hat{\sigma }$ ($10^{-4}$).

ABEP		BEGG		RS		ESC		ASL	
$\hat{Var}\left(\hat{\mu }\right)$	$\hat{Cov}\left(\hat{\mu },\hat{\sigma }\right)$	$\hat{Var}\left(\hat{\mu }\right)$	$\hat{Cov}\left(\hat{\mu },\hat{\sigma }\right)$	$\hat{Var}\left(\hat{\mu }\right)$	$\hat{Cov}\left(\hat{\mu },\hat{\sigma }\right)$	$\hat{Var}\left(\hat{\mu }\right)$	$\hat{Cov}\left(\hat{\mu },\hat{\sigma }\right)$	$\hat{Var}\left(\hat{\mu }\right)$	$\hat{Cov}\left(\hat{\mu },\hat{\sigma }\right)$
$\hat{Cov}\left(\hat{\mu },\hat{\sigma }\right)$	$\hat{Var}\left(\hat{\sigma }\right)$	$\hat{Cov}\left(\hat{\mu },\hat{\sigma }\right)$	$\hat{Var}\left(\hat{\sigma }\right)$	$\hat{Cov}\left(\hat{\mu },\hat{\sigma }\right)$	$\hat{Var}\left(\hat{\sigma }\right)$	$\hat{Cov}\left(\hat{\mu },\hat{\sigma }\right)$	$\hat{Var}\left(\hat{\sigma }\right)$	$\hat{Cov}\left(\hat{\mu },\hat{\sigma }\right)$	$\hat{Var}\left(\hat{\sigma }\right)$
1.3731	0.0919	0.0602	3.2295 × $10^{-4}$	0.0317	-0.1177	3.1921	1032	344.4	7.592
	0.2517		0.0901		0.0085		3747		0.6688

**Table 7:** Counted data at ranges [−10, −0.4, −0.2, 0, 0.2, 0.4, 10].

Real Data	0	9	45	62	2	0	0
ABEP	0	8	46	60	4	0	0
BEGG	0	11	44	59	4	0	0
ESC	0	8	52	54	4	0	0

ABEP: Asymmetric bimodal exponential power; BEGG: Bimodal extended generalized gamma; ESC: Exponentiated sinh Cauchy.

## Abbreviations

   The following abbreviations are used throughout the text:

PDF	Probability density function
CDF	Cumulative density function
ML	Maximum likelihood
NC	Normalizing constant
GOFT	Goodness of fit test
RCLBs	Rao-Cramér lower bounds
ABEP	Asymmetric bimodal exponential power
BEP	Bimodal exponential power
EP	Exponential power
BEGG	Bimodal extended generalized gamma
RS	Rathie-Swamee
ESC	Exponentiated sinh Cauchy
ASL	Alpha–skew–Laplace
HGA	Hybrid genetic algorithm
KS	Kolmogorov–Smirnov
AD	Anderson–Darling
CVM	Cramér–von Mises
GG	Generalized gamma

## Appendix A. Random Number Generation Procedure from ABEP Distribution

**Algorithm 1** Random Number Generation Procedure from ABEP Distribution
1: **FOR** *i* FROM TO the number of sample size $n_{1}$ from $\alpha_{1}$, 2: $v_{1}=k_{1}\left(1-\varepsilon \right)$, 3: Generate *y* from Gamma distribution with parameters $\frac{\delta_{1}+1}{\alpha_{1}}$ and 1, 4: $x_{1}=\mu +\sigma v_{1}y^{1/\alpha_{1}}$. 5: **END FOR** 6: **FOR** *i* FROM TO the number of sample size $n_{0}$ from $\alpha_{0}$, 7: $v_{0}=k_{0}\left(1+\varepsilon \right)$, 8: Generate *y* from Gamma distribution with parameters $\frac{\delta_{0}+1}{\alpha_{0}}$ and 1, 9: $x_{0}=\mu +\sigma v_{0}y^{1/\alpha_{0}}$. 10: **END FOR** 11: Let *x* be a row vector with $n=n_{1}+n_{0}$ elements of two vectors $x_{1}$ with $n_{1}$ for negative data and $x_{0}$ with $n_{0}$ for positive data, that is, $x_{n}=\left(x_{1},x_{0}\right)$.

## Appendix B. Real Data Sets Used in Analysis Procedure

**Table A1:** Homo sapiens Pig7 (PIG7) mRNA, complete cds Chr.16 [381663, (EW), 5’:AA059047, 3’:AA059031], n=118.

0.177	0.061	−0.069	−0.162	−0.114	0.13	0.305	0.172	0.154	−0.073	−0.135	−0.089	−0.16	−0.161	0.124
0.007	0.013	−0.081	−0.049	−0.07	0.122	0.095	−0.061	−0.077	0.128	0.203	0.045	−0.065	−0.123	−0.207
−0.217	−0.006	−0.089	−0.053	−0.208	−0.101	−0.157	−0.175	−0.061	0.004	−0.047	−0.021	−0.046	−0.233	−0.049
0.159	0.171	0.076	−0.112	−0.101	−0.107	−0.127	−0.253	0.07	0.111	0.053	0.107	−0.05	0.004	−0.06
0.022	0.193	0.071	0.02	0.015	−0.016	0.064	−0.119	0.123	0.046	−0.126	−0.086	−0.171	−0.091	−0.013
−0.083	−0.077	−0.164	0.054	0.133	0.153	0.194	0.351	0.309	0.331	0.328	−0.093	0.298	0.112	0.107
0.22	0.406	0.171	0.255	0	0.302	0.146	0.294	0.27	0.157	0.102	0.375	0.076	0.206	0.253
0.067	0.256	0.044	0.124	0.427	0.116	−0.097	0.108	−0.036	0.113	0.003	0.1	0.204

**Table A2:** SID 377353, ESTs [5’:, 3’:AA055048], n=118.

0.029	0.062	0.011	0.009	0.065	−0.128	0.133	0.116	0.184	0.111	−0.066	−0.049	0.05	0.137	0.162
0.173	0.033	0.107	0.11	0.147	0.118	0.172	0.284	−0.137	0.038	−0.145	−0.181	−0.155	0.198	0.024
0.079	−0.252	0.062	0.097	0.032	0.026	0.195	0.019	0.138	−0.3	−0.105	−0.11	−0.168	−0.173	−0.15
0.078	0.113	−0.047	0.024	0.001	−0.075	0.014	0.058	−0.083	−0.339	−0.177	−0.073	−0.044	−0.106	−0.159
−0.101	−0.074	−0.126	−0.131	−0.22	−0.184	−0.105	0.173	0.151	0.064	−0.007	−0.005	−0.189	−0.219	−0.301
−0.212	−0.088	0.157	0.042	0.184	0.114	0.102	0.119	−0.064	−0.075	0.073	0.038	0.017	−0.134	−0.118
−0.097	0.059	0.025	−0.102	−0.096	−0.035	0.057	−0.055	0.015	−0.23	−0.115	0.255	0.034	0.078	0.129
0.081	0.032	0.047	−0.145	0.012	−0.224	0.074	−0.06	−0.137	0.034	0.009	−0.139	−0.141
